# Network Analysis on the Symmetric Coordination in a Reinforcement-Learning-Based Minority Game

**DOI:** 10.3390/e27070676

**Published:** 2025-06-25

**Authors:** Chunqiang Shao, Wenjia Rao, Wangfang Xu, Longbao Wei

**Affiliations:** 1 School of Sciences, Hangzhou Dianzi University, Hangzhou 310018, China; 2 School of Accounting, Zhejiang University of Finance and Economics, Hangzhou 310018, China; 3 China Academy for Rural Development, School of Public Affairs, Zhejiang University, Hangzhou 310058, China

**Keywords:** minority game, reinforcement learning, complex network

## Abstract

The Minority Game (MG) is a paradigmatic model in econophysics, widely used to study inductive reasoning and self-organization in multi-agent systems. Traditionally, coordinated phases in the MG are associated with spontaneous symmetry breaking, where agents differentiate into polarized roles. Recent work shows that policy-based reinforcement-learning can give rise to a new form of symmetric coordination—one achieved without role segregation or strategy specialization. In this study, we thoroughly analyze this novel coordination using tools from complex networks. By constructing the correlation networks among agents, we carry out a structural, functional, and temporal analysis of the emergent symmetric coordination. Our results confirm the preservation of symmetry at the collective level, and reveal a consistent and robust form of distributed coordination, demonstrating the power of network-based approaches in understanding the emergent order in adaptive multi-agent systems.

## 1. Introduction

Game theory traditionally assumes fully rational agents capable of optimizing their actions based on strategic foresight and deductive reasoning. However, the Minority Game (MG), initially introduced by Challet and Zhang as a formalized version of the El Farol Bar Problem [1,2], presents a contrasting framework where agents adapt solely based on past outcomes without predicting future rewards. Originating from econophysics, MG has not only become a representative model for studying coordination in competitive, resource-limited systems, but also evolved into a classic case of interdisciplinary research, bridging physics, economics, and social science. Over the years, the interplay between these disciplines has driven MG from a basic resource allocation model to a versatile framework incorporating evolutionary strategies, agent heterogeneity, and network structures, substantially enriching our understanding of decentralized coordination in multi-agent systems [3,4,5,6,7,8,9,10].

In recent years, reinforcement learning (RL) has been applied to MG to model adaptive agent behavior in dynamic environments [11,12,13,14,15]. Among these approaches, Q-learning, a value-based algorithm that estimates expected future rewards, has been most widely used to reduce volatility and improve resource allocation. However, such methods often lead to symmetry breaking, resulting in role polarization among agents in a manner similar to the crowd–anticrowd mechanism in the standard MG [16,17,18,19]. In a more recent work [20], an algorithm from policy-based RL—another category of RL that directly updates policies without estimating action values [21,22]—has been applied to the MG. Due to the smooth adaptation dynamics of this algorithm, a new type of coordination was observed, one that does not involve symmetry breaking. In this novel coordination phase, each agent remains stochastic at the individual level, yet a form of implicit “mutual understanding” emerges at the collective level, leading to overall coordination without explicit role polarization [20].

This novel coordination phase demonstrates how stochastic individual behaviors can collectively form coherent patterns, representing a new type of coordination mechanism with potential universality in complex systems. However, while the work [20] has established the existence of this novel phase and characterized it through various macro- and microscopic measures, several important aspects remain unexplored. For example, how exactly are agents structurally connected in this coordination phase? How do these connections evolve over time? What functional roles do these connections play, and how robust are they under external attacks? A comprehensive characterization of these features is crucial for a deeper understanding of this novel coordination phase, and thus serves as the primary objective of this study.

In the present work, we employ the tool of complex network analysis [23,24] to thoroughly investigate this novel symmetric coordination phase. For reasons detailed in Section 3, we partition the correlation network into positive and negative subnetworks and analyze them separately. Our findings indicate that the coordination emerging from policy-based reinforcement learning is not only symmetric in behavior but also structurally balanced and functionally robust. The resulting correlation networks exhibit distributed connectivity, moderate clustering, and a high tolerance to perturbations, without signs of role polarization or hierarchical concentration. The positive subnetwork is organized and redundant, while the negative subnetwork remains sparse and weakly structured, yet it contributes to a global coordination state. Additionally, we quantitatively characterize the temporal evolution of these networks, revealing how the structure and functional patterns develop and stabilize over time. These findings confirm that the observed symmetry-preserving coordination is not merely a statistical artifact but is embedded in the underlying network architecture, where balanced connectivity and distributed organization jointly sustain coherent coordination without explicit role differentiation. This underscores how even in the absence of centralized control, stochastic behaviors can self-organize into robust, functionally integrated network architectures.

This paper is organized as follows. In Section 2, we briefly review the reinforcement-learning-based Minority Game, focusing on the policy-based learning mechanism and the key behavioral observations. In Section 3, we present the construction of the coordination network with a properly chosen threshold. Section 4, Section 5 and Section 6 present a detailed analysis of the coordination phase using complex network methods, covering its structural properties, functional characteristics, and temporal evolutions. Finally, in Section 7 we offer concluding remarks and discuss potential directions for future work.

## 2. Model and Behavioral Characterization

We begin with briefly outlining the reinforcement-learning setup in this section; the full implementation details are provided in [20]. We consider the classic MG model, which is a repeated N-player game played over multiple rounds. In each round, a population of N agents makes binary decisions at each discrete time step: action a=1 (e.g., attend the bar) or action a=0 (e.g., stay at home). Agents whose choices belong to the global minority group receive a unit reward. These rewards are accumulated across multiple rounds to determine the agents’ incomes. All agents update their choices synchronously at each time step. A key macroscopic observable is the *attendance* A(t), defined as the number of agents choosing action 1 at time *t*. Clearly, the system achieves its maximum total reward when A(t)≈N/2, i.e., when the population splits evenly. The system’s coordination efficiency is characterized by the *normalized volatility*

(1) $$\sigma^{2}/N=\frac{1}{N}\left[\langle A{\left(t\right)}^{2}\rangle -{\langle A\left(t\right)\rangle }^{2}\right],$$

where 〈·〉 denotes a time average taken in the steady state. Lower volatility indicates better coordination, and $\sigma^{2}/N=0.25$ serves as a benchmark value corresponding to agents making independent random choices (in some literature, the binary actions are denoted by a=±1 for attending the bar or not. In this case, the normalized volatility will be $\sigma^{2}/N=1$ for random choices [25]. Apparently, these two choices are equivalent in essence).

During the game, each agent observes a fixed-length history of past winning outcomes, referred to as the memory length *M*, and makes independent decisions based on this information. In the original MG, agents rely on fixed strategies selected from a predefined pool. However, RL algorithms such as Q-learning allow agents to dynamically update their strategies based on accumulated experience. In this study, we follow the policy-based approach introduced in [20], where each agent’s strategy is represented as a parameterized stochastic policy. Specifically, the policy $\pi_{i}\left(a|s\right)$ is expressed as a softmax function:

(2) $$\pi_{i}\left(a|s;\theta_{i},\tau \right)=\frac{\exp(\theta_{i,s,a}/\tau )}{\sum_{a^{\prime }\in \left\{0,1\right\}}\exp\left(\theta_{i,s,a^{\prime }}/\tau \right)}$$

Here, $\theta_{i,s,a}$ is the agent’s preference for action *a* in state *s*, and τ is a temperature parameter that controls the trade-off between exploitation and exploration: a lower τ makes the agent more likely to exploit the most preferred action that makes the policy more deterministic, while a higher τ promotes exploration by assigning more similar probabilities to all actions. This softmax formulation is mathematically equivalent to the logit rule widely used in noisy best-response dynamics in evolutionary game theory, and is closely related to the Glauber dynamics commonly used in statistical physics.

Each agent’s policy is updated through the REINFORCE algorithm, in which the agent continuously interacts with the environment by executing episodes of length $T_{ep}$. Within one episode, each agent participates in $T_{ep}$ rounds of the game using their own strategy, receiving a reward of 1 if it is in the minority group (and 0 otherwise) at each round. The agent then computes a return $G_{i}\left(t\right)$ by summing its rewards over the episode, which serves as the learning signal. This $G_{i}\left(t\right)$ is then used to update the policy parameters as follows:

(3) $$\theta_{i,s_{t},a}\leftarrow \theta_{i,s_{t},a}+\alpha G_{i}\left(t\right)\nabla_{\theta_{i,s_{t},a}}\log\pi_{i}\left(a_{i}\left(t\right)|s_{t}\right)$$

where the learning rate is set to be α=0.001 in this study. This update rule adjusts the probability distribution over actions in a way that increases the likelihood of actions that have historically led to higher cumulative rewards. Importantly, this policy-based update scheme is inherently smooth, maintaining stochastic behavior while preventing premature convergence to polarized strategies.

**Figure 1:**
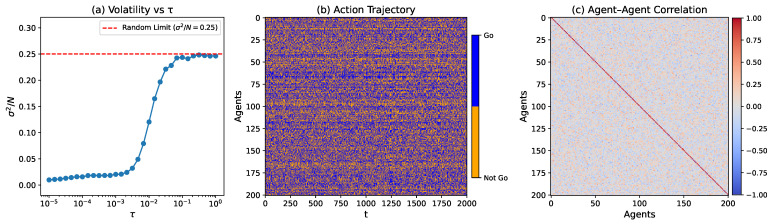
(**a**) The normalized volatility $\sigma^{2}/N$ as a function of temperature τ for N=201 and M=5. The red dashed line marks the benchmark volatility $\sigma^{2}/N=0.25$, corresponding to random choice behavior. The system reaches a well-coordinated state at a low temperature, indicated by suppressed volatility. (**b**) Agent action trajectories at $\tau =10^{-3}$, showing dynamic activity without any persistent polarization. (**c**) Agent–agent correlation matrix calculated from the action trajectories, where positive correlations (red) indicate similar behavior patterns, while negative correlations (blue) indicate opposing behaviors. The matrix reveals a distributed coordination structure despite the apparent randomness in individual agent trajectories.

The simulation results for different population sizes and memory lengths have been thoroughly discussed in [20], and here—without loss of generality—we focus on the case with N=201 and M=5 (the population size here (N=201) is larger than the one discussed in [20] (N=101) and most MG studies, which is because the subsequent network analysis requires more nodes. Besides, the coordination pattern studied below is robust with varying *M* [20], and here M=5 is selected as a representative case). Figure 1a shows the volatility $\sigma^{2}/N$ as a function of the temperature-like parameter τ. We clearly observe a wide regime $\tau \in [10^{-5},\;5\times 10^{-3}]$ with a volatility significantly below the random benchmark, indicating a well-coordinated phase. In Figure 1b, we plot the action trajectories of all agents over the final 2000 training steps for a representative temperature ($\tau =10^{-3}$) in this low-volatility coordination regime. We can see that individual agent trajectories appear disordered, exhibiting no evident role separation or long-term specialization, which suggests the absence of global symmetry breaking. However, despite the apparent randomness at the individual level, distributed correlations among agents do exist, as revealed by the Pearson correlation matrix shown in Figure 1c. This correlation structure indicates that the observed coordination emerges not from individual stability but from network-level interdependencies.

Previous work [20] has characterized this emergent coordination phase using macroscopic measures and microscopic statistical indicators, confirming the absence of symmetry breaking. However, several critical questions remain unanswered: How is this symmetry-preserving coordination internally structured? How does it evolve over time? And to what extent is it robust against external perturbations? Addressing these questions is the primary objective of this study. Figure 1b,c clearly suggest that the observed coordination is not rooted in individual-level order but rather in the extensive correlations established among agents. Consequently, complex network analysis emerges as the most suitable framework to systematically characterize this underlying structure. Accordingly, in the following sections, we will employ network-based approaches to examine the coordination phase from structural, functional, and temporal perspectives. Without loss of generality, we will focus on the representative case with $\tau =10^{-3}$; other cases in the coordination phase exhibit qualitative similar results.

## 3. Constructing Signed Coordination Network

To investigate the structural organization underlying the observed coordination phase, the first step is to construct a network representation based on the statistical correlations among agents’ action sequences. A natural way to build the correlation network is to treat the Pearson matrix as the adjacent matrix, where each node represents an agent and each edge represents pairwise correlation. However, as shown in Figure 1c, both positive and negative correlations are present, each carrying distinct behavioral implications in the context of MG: Positive correlations suggest that agents tend to make similar decisions over time, potentially competing for the same resource; by contrast, negative correlations indicate anti-aligned behaviors, where agents adopt opposing actions. The latter pattern is particularly significant, as it avoids crowding and is in agreement with MG’s rules. Thus, strong negative correlations may reflect not mere opposition but a form of adaptive coordination.

Given this behavioral asymmetry, treating the correlation network as a single, unsigned network would obscure these distinct modes of interaction. Instead, we adopt a *signed network* approach, decomposing the correlation matrix into two subnetworks: one capturing positively correlated agent pairs and the other capturing negatively correlated pairs. This separation allows us to systematically analyze how cooperative and competitive interactions contribute to the overall network structure and the maintenance of coordination without role specialization.

An essential step in constructing the positive and negative subnetworks is to determine appropriate correlation thresholds that effectively distinguish significant interactions from noises. Small correlation values may arise from random fluctuations rather than stable interaction patterns, and retaining such spurious edges would obscure the true network structure. To address this, we adopt a data-driven procedure that systematically examines how key structural metrics evolve as the threshold value varies.

We assess three key metrics to guide the threshold selection: the number of edges, the average degree, and the number of connected components. The number of edges, including positive and negative edges separately, indicates how densely the network is connected. The average degree measures the typical number of connections per node, which is expected to decrease as the threshold increases. The number of connected components reveals how the network fragments when weaker correlations are pruned. By tracking these metrics, we can identify a threshold that maintains sufficient connectivity while preventing the network from becoming overly dense or excessively fragmented.

**Figure 2:**
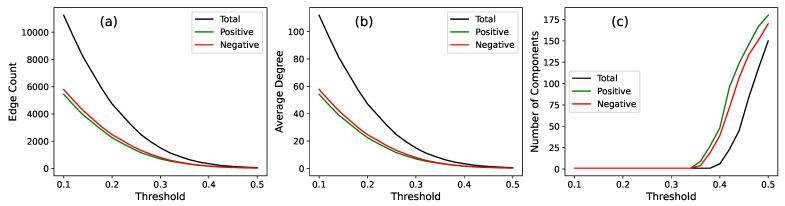
Threshold scan results of network structural metrics (edge count, average degree, and connected components) as a function of the threshold *t*, where the total network and positive/negative sub-networks are calculated separately. While the edge count and average degree decrease steadily with an increasing threshold, there is a sharp rise in the number of connected components around t=0.35, marking a critical point where network fragmentation becomes pronounced.

Figure 2 presents the results of scanning the threshold *t* across a range of values, illustrating how the network structure evolves as weaker correlations are pruned. As expected, the number of edges decreases with an increasing threshold, with both positive and negative edges exhibiting a declining trend. The average degree follows a similar pattern, reflecting a progressively sparser network structure. By contrast, the number of connected components shows a more pronounced behavior, remaining relatively stable at lower thresholds but rising sharply around t=0.35 (we have performed the same threshold scanning in the cases with N=101 and, curiously, found the same critical value, which suggest that it may not merely be a numerical coincidence. This certainly deserves further exploration, which is, however, beyond the scope of the present work). This sharp increase indicates a critical point where the network begins to fragment significantly, suggesting that the structure becomes increasingly disconnected. Based on these observations, we select a threshold t=0.3, as it balances network sparsity and connectivity while preventing excessive fragmentation. This threshold will be applied separately to the positive and negative subnetworks to capture distinct interaction patterns in the subsequent analysis.

## 4. Structural Analysis

**Figure 3:**
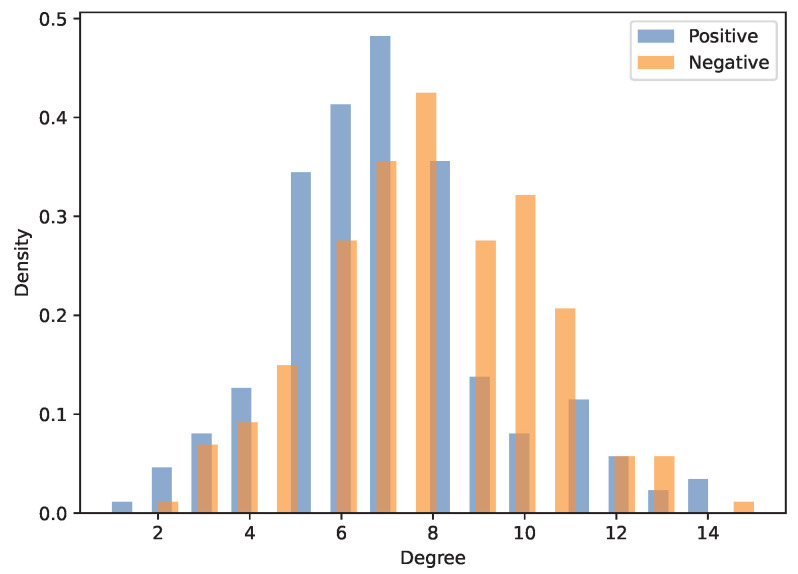
Degree distributions of the positive and negative networks. Both distributions are unimodal, with the negative network displaying a slightly broader spread, reinforcing the observed difference in average degree.

To establish a structural baseline for the subsequent analyses, we first computed several fundamental topological network metrics, including the number of nodes, the number of edges, and the average degree [26]. As it turns out, both subnetworks contain 201 nodes, ensuring an identical network size across both interaction types. However, the number of edges differs notably: the positive network has 699 edges, whereas the negative network has 804 edges. Consequently, the average degree in the positive network is 6.96, while the negative network has a slightly higher average degree of 8.00. This discrepancy indicates a denser connection structure in the negative network, suggesting that agents are more frequently involved in interactions characterized by opposing strategies. To be complete, the degree distributions of both networks are presented in Figure 3. Both distributions are unimodal and nearly symmetric, lacking heavy-tailed or power-law characteristics. The primary observation is that the negative network exhibits a slightly broader spread and a higher frequency of high-degree nodes, reinforcing the earlier finding of a denser connectivity pattern.

Next, we evaluated the networks’ structural connectivity and path efficiency by calculating the number of connected components, the size of the largest component, and the average path length. Both networks consist of a single connected component encompassing all 201 nodes, ensuring that all agents are part of a single interaction network. The average path length is slightly shorter in the negative network (2.81) compared to the positive network (3.07), indicating more direct connections and potentially faster information transmission in the negative network. This aligns with the earlier observation of a higher average degree, suggesting that interactions among agents adopting opposing strategies are more frequent and structurally efficient.

The size of the k-core further highlights the structural differences between the networks [27]. The positive network has 166 core nodes, whereas the negative network contains 185, suggesting a more extensive core of highly interconnected agents in the negative network. This structure may serve as a backbone for sustaining antagonistic strategies, providing a stable framework for frequent oppositional interactions. Additionally, the assortativity values are near zero for both networks (0.0102 for the positive and 0.0267 for the negative), indicating that connections are not significantly influenced by the node degree. This further suggests that the observed structural differences are primarily driven by interaction patterns rather than degree correlations.

Following the analysis of basic structural parameters, we examined the networks’ local structural patterns through clustering coefficient and motif analysis. The clustering coefficient is 0.0894 for the positive network and 0.0003 for the negative network, indicating a pronounced disparity in local clustering. The positive network exhibits a tendency for agents to form tight-knit clusters, potentially representing small collaborative subgroups where agents display similar behavior. By contrast, the negative network remains largely devoid of such localized cohesion, aligning with its more fragmented and adversarial structure. To further quantify the variability in local clustering, we calculated the entropy of the clustering coefficient distribution, which is 4.268 for the positive network and only 0.112 for the negative network. This stark contrast indicates that while the positive network supports diverse local cohesion patterns, the negative network is characterized by uniformly sparse and structurally homogeneous interactions.

To further investigate these local structural differences, we conducted a motif analysis focused on three-node subnetworks, which serve as elementary building blocks for capturing recurring interaction patterns within the network [28]. Specifically, we examined the occurrence of fully connected triads (three-edge motifs), which indicate tightly closed group interactions and potential coordination clusters. The statistical significance of these motifs was assessed using Z-scores, which quantify to what extent does the observed motif frequency deviate from a randomized baseline with the same degree of distribution. In the context of MG systems, such motif patterns provide insights into how localized coordination or opposition strategies manifest in small-scale interaction clusters. The results reveal a striking asymmetry: the positive network contains 214 such motifs with a Z-score of 36.00, indicating significant over-representation relative to random expectation. By contrast, the negative network exhibits only one three-edge motif, with a Z-score of -7.03, underscoring a substantial under-representation. This disparity illustrates the distinct structural roles of positive and negative links—while positive links facilitate closed-loop group interactions, negative links actively suppress them, resulting in a more fragmented and less cohesive local structure.

Having characterized the basic structural parameters and local interaction patterns, we now extend the analysis to the community level via the Louvain method to understand how agents organize into cohesive or fragmented groups. Community analysis detects clusters of nodes that are more densely connected internally than externally, providing a perspective on how local interactions aggregate into larger structural patterns within the networks [29]. The results show that both networks exhibit similar basic community metrics, with 10 and 9 communities in the positive and negative subnetworks, respectively, and comparable maximum (32 vs. 36 nodes) and minimum (9 nodes in both) community sizes. The entropy values, measuring the variability in community size, are also close (0.4229 vs. 0.3966). Thus, in terms of basic partition structure, the two networks do not differ substantially.

However, deeper differences emerge when considering how these communities are internally structured and externally connected. The positive network shows a higher modularity score (0.4491 vs. 0.3381), indicating more clearly defined, internally focused clusters. This pattern is reinforced by community cohesion, which averages 0.2704 in the positive network but remains zero in the negative network, suggesting that positive interactions promote tightly knit subgroups, whereas negative interactions do not. Community density further underscores this contrast: the positive network exhibits lower external-to-internal connectivity ratios (1.7981 vs. 2.5173), reflecting more insular, cooperative clusters. By contrast, the negative network is characterized by fragmented, outwardly connected groups, which is consistent with more competitive or antagonistic dynamics.

Overall, while the basic community structure appears similar across both networks, the nature of internal coordination and external connectivity diverges sharply. Positive interactions facilitate cohesive, internally connected clusters, while negative interactions give rise to fragmented, outwardly connected groups, aligning with the broader pattern of adversarial dynamics observed in the previous analysis.

For convenience, we have summarized all of these topological metrics in Table 1. The structural analysis highlights how cooperative and antagonistic strategies manifest distinctly in the MG framework. Positive interactions form stable, cohesive clusters characterized by localized reinforcement, as reflected in higher clustering, abundant triadic motifs, and an internally focused community structure. Conversely, negative interactions result in dispersed, outwardly connected patterns, lacking internal cohesion and forming more fragmented, externally oriented groups. These structural differences indicate that while cooperative strategies consolidate locally, antagonistic strategies distribute more diffusely, aligning with a broader pattern of dispersed opposition within the MG context.

## 5. Functional Properties and Robustness

Having examined the structural properties of the positive and negative networks, we now turn to their functional characteristics. While structural metrics describe how the network is built, functional metrics aim to reveal how effectively it supports coordination, adaptation, and information propagation—key aspects in multi-agent systems like MG.

We focus on *global efficiency*, *local efficiency*, and *closeness centrality*, as these metrics collectively capture the system’s capacity for both local resilience and global information flow [26]. The negative network exhibits slightly higher global efficiency (0.3897 vs. 0.3601), indicating a more compact structure that facilitates rapid information transmission across the entire network. This aligns with its shorter path length, reflecting a direct and dispersed connection pattern that is conducive to the competitive nature of MG, where rapid responses to global signals are advantageous. By contrast, the positive network shows markedly higher local efficiency (0.1892 vs. 0.0003), indicating stronger local clustering and redundancy. This suggests that positively correlated agents form tightly knit clusters, but such localized coherence may not effectively support the strategic divergence required in the MG dynamics. Additionally, the negative network has higher average closeness centrality (0.3576 vs. 0.3274), reinforcing its role as a globally integrated structure where nodes are more centrally positioned to access information rapidly. We also checked the rich-club coefficient and found minimal organization in both networks, indicating that high-degree nodes do not form a tightly connected core.

Overall, the functional analysis suggests that while the positive network leverages local redundancy to maintain cluster cohesion, the negative network prioritizes global connectivity, enabling rapid information diffusion and strategic responsiveness. This contrast underscores the divergent strategic roles of local vs. global structures in the MG, where localized clusters may reinforce cooperative behavior, while globally dispersed connections facilitate competitive interactions.

To further understand how each network responds to external perturbations, we analyzed their *robustness* under node removal. This analysis is particularly relevant in the MG framework, where strategic interactions may selectively target key nodes or disrupt coordinated clusters [30,31]. We considered two standard attack scenarios: *targeted attacks*, simulating the removal of high-degree nodes to weaken dominant structures, and *random attacks*, representing non-strategic perturbations. Since positive and negative networks have different meanings in the MG context, and thus we will study their robustness separately. Specifically, we tracked three indicators as functions of the fraction of removed nodes: the size of the largest connected component (LCC), the global efficiency, and the average shortest path length.

**Figure 4:**
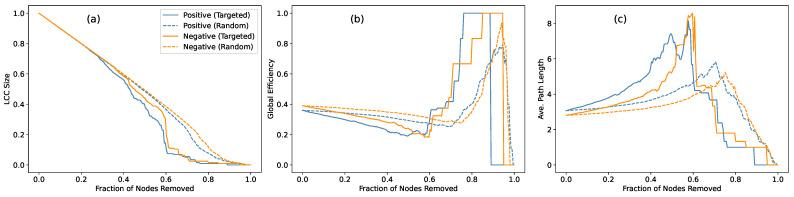
Robustness of the positive and negative networks under two types of attacks: targeted attack and random attack. (**a**) Size of the largest connected component (LCC); (**b**) global efficiency; (**c**) average path length within the largest component.

The simulation results are presented in Figure 4, where all curves eventually converge toward network collapse as more nodes are removed—a natural outcome given the extensive structural disintegration. However, more informative differences emerge in the early and intermediate regimes of the attack. During these stages, the LCC size remains relatively similar across both networks, indicating comparable resilience in preserving basic connectivity. Yet, the global efficiency and average path length reveal more distinct patterns: the negative network consistently maintains higher efficiency and shorter paths under both attack scenarios. This suggests that the negative network’s structure is less reliant on specific nodes for maintaining communication pathways, implying an absence of central hubs that could critically disrupt connectivity when removed.

The contrast between targeted and random attacks further underscores this point. As expected, targeted attacks degrade performance more rapidly, particularly in networks with well-defined hub nodes. Yet, the negative network continues to sustain its efficiency advantage, suggesting that its structure is more evenly distributed, with no single node exerting critical control over the entire network. This interpretation is further supported by the random attack results, where the impact on both networks is relatively modest, indicating that neither network is particularly hub-centric. We also tested a larger network with N=401 nodes, where the same patterns persisted, though structural collapse occurred at slightly later removal fractions. These results confirm that the observed robustness patterns are not merely size-dependent but reflect intrinsic organizational differences between the two networks.

These findings provide additional insight when interpreted in the context of MG dynamics. The negative network, characterized by its decentralized, hub-less architecture, consistently preserves global efficiency and maintains short path lengths even under targeted attacks, effectively mitigating the impact of node removal through a dispersed, functionally robust structure. This pattern aligns with MG’s competitive strategy, where rapid, distributed information spread reduces the dependence on key nodes and prevents structural collapse.

## 6. Temporal Evolution Analysis

While previous structural and functional metrics provide a static snapshot of the coordination phase, a more complete understanding requires examining how these network properties evolve over time [32]. To this end, we now turn to the temporal evolution of the network and collective behavior. This dynamic analysis serves two purposes: to verify that the system converges to a stable coordination regime after sufficient training, and to examine how key topological and functional properties evolve over time.

For our purpose, we divide the entire training process into non-overlapping sliding windows of fixed length (1000 time steps per window), and in each window, we reconstruct the positive and negative correlation subnetworks based on the Pearson correlation matrix. To capture key structural and functional changes over time, we focus on three representative metrics: (i) the average degree, reflecting overall network density, (ii) the average path length, indicating communication efficiency and diffusion patterns, and (iii) modularity (via the Louvain method), identifying shifts in community structure. Other metrics, such as the global efficiency, clustering coefficient, closeness centrality, and local efficiency, were calculated but omitted due to either trivial constancy or information redundancy. Additionally, to examine whether there is any systematic symmetry breaking among agents, we define the net preference bias for each time step *t* as

(4) $$m\left(t\right)=\frac{2}{N}\sum_{i=1}^{N}a_{i}\left(t\right)-1,$$

where $a_{i}\left(t\right)\in \left\{0,1\right\}$ represents the action taken by agent *i* at time *t*. The bias quantifies the overall preference balance, with values close to zero indicating no dominant preference or symmetry breaking across the agent population.

**Figure 5:**
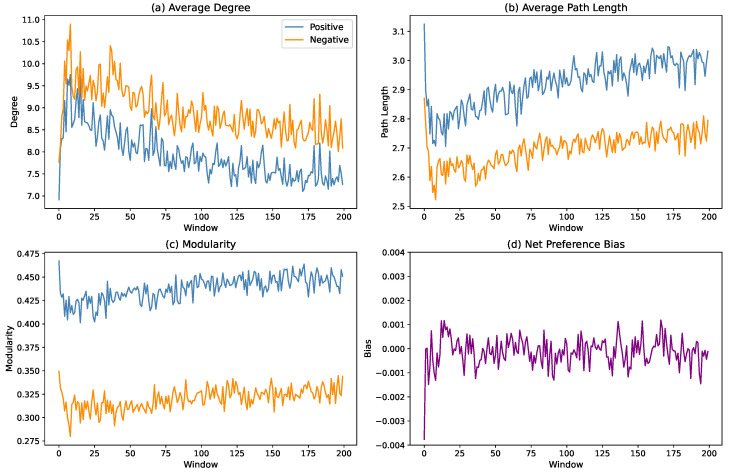
Temporal evolution of key network metrics over successive windows of training, each corresponding to 1000 learning steps. Panels (**a**–**c**) show the average degree, average path length, and community modularity for the positive (blue) and negative (orange) correlation subnetworks, respectively; (**d**) shows the net preference bias, which remains centered on zero throughout, indicating the absence of symmetry breaking.

Figure 5 shows the temporal trajectories of these four metrics across all windows. All three network metrics (average degree, path length, and modularity) converge within the 50–75 window range, indicating that the policy-gradient learning process successfully drives the system toward a stationary coordination phase. In this state, the negative subnetwork consistently exhibits higher density and shorter path lengths compared to the positive subnetwork, suggesting a more integrated and compact network structure. By contrast, the positive subnetwork maintains higher modularity, reflecting a more localized, community-oriented organization. Notably, the net preference bias remains near zero throughout the entire training process, indicating that the system never develops any significant behavioral polarization, thus preserving symmetry at the global level.

More insights can be gained by evaluating the evolution process in greater detail. For the three network metrics, the training divides into two distinct stages. During windows 0–20, the average degree increases rapidly as new connections form, leading to a decrease in path length, demonstrating the increasing associations among agents. Meanwhile, modularity decreases, indicating that these new connections primarily link nodes across different communities, diluting existing community boundaries. This reflects an exploratory phase, where agents establish broad, cross-community connections to maximize information access. After window 20, as the learning process stabilizes and agents begin to identify more reliable connections, the network enters a prolonged pruning phase, selectively removing less consistent links while retaining stronger intra-community connections. This consolidation process leads to a slight decrease in the average degree, a marginal increase in the path length, and a rise in modularity. After approximately 100 windows, the network structure stabilizes.

By contrast, the net preference bias remains close to zero throughout the entire process, showing no indication of non-monotonic behavior. This rapid stabilization can be attributed to the inherent nature of the policy-gradient method, which employs probabilistic policy updates aimed at maintaining global symmetry rather than enforcing specific roles or polarizations. As a result, the system converges almost immediately to a state of balanced coordination, effectively preventing the emergence of any systematic bias from the very beginning.

## 7. Conclusions and Discussion

This work presents a systematic investigation into the novel symmetry-preserving coordination phase in the reinforcement-learning based minority game, a distinctive form of collective coordination characterized by the absence of role polarization and explicit strategy differentiation. By employing complex network analysis, this work offers an essential and previously missing perspective on how decentralized coordination emerges and stabilizes, complementing basic behavioral observations with a detailed characterization of the underlying interaction architecture.

Specifically, we partitioned the correlation network into positive and negative subnetworks and analyzed their structural and functional characteristics—including connectivity patterns, clustering properties, and robustness to perturbations—along with their temporal evolution. The analysis reveals a clear functional asymmetry: the negative subnetwork, though sparse and weakly structured, captures the core strategic mechanism of the Minority Game by supporting anti-correlated agent behaviors that reduce overcrowding and suppress role polarization, thus playing an indispensable role in the formation of decentralized coordination. By contrast, the positive subnetwork exhibits higher density and local redundancy, reflecting spontaneous subgroup coherence and short-range behavioral alignment. Together, these two subnetworks form a complementary structure: the negative links facilitate global coordination through behavioral divergence, while the positive links provide local stability and cohesion. Temporal analysis further confirms the stabilization of these patterns, showing how initial stochastic fluctuations consolidate into robust, distributed coordination structures.

Our findings offer deeper theoretical insight into how decentralized agent strategies can spontaneously form stable and coherent coordination structures without explicit role differentiation. From a statistical physics perspective, this phenomenon can also be interpreted in terms of frustration: the minority constraint in the game induces unavoidable conflicts among agents’ objectives, similar to the frustration observed in spin glass systems. As highlighted by Cavagna [33], such frustrated environments often lead to rich collective behavior. Our results suggest that the symmetry-preserving, distributed coordination uncovered here represents a collective mechanism for alleviating frustration through adaptive stochastic strategies. Taken together, these insights clarify the essential link between network structure and emergent coordination, and point toward a broader principle whereby distributed interactions inherently facilitate self-organization—a conceptual framework that may be widely relevant to understanding complex adaptive phenomena in decentralized systems.

Last but not least, the methodological combination of policy-based reinforcement learning and complex network analysis demonstrated here shows substantial promise beyond the Minority Game context. This integrated approach could be effectively applied to analyze emergent coordination and self-organization phenomena in systems such as social influence networks, opinion dynamics, cooperative behavior in socio-economic systems, ecological interaction networks, and statistical physics models of collective behavior, enabling a deeper understanding of how global order spontaneously arises from local feedback interactions. In addition, the structural patterns uncovered in this work raise an intriguing possibility for future research: if similar network structures were constructed in advance and the agent behavior assigned accordingly, could comparable coordination outcomes be achieved? This reverse perspective would not only complement the current bottom–up analysis, but also underscore the potential of complex network analysis—not just as a diagnostic tool, but as a means to reveal and even inform the design of coordination mechanisms.

## Figures and Tables

**Table 1:** Summary of network structural metrics for the positive and negative networks, with key differences highlighted in bold.

Metric	Positive Network	Negative Network
num_nodes	201	201
num_edges	**699**	**804**
avg_degree	**6.96**	**8.00**
avg_path_length	3.07	2.81
connected_components	1	1
largest_component_size	201	201
k_core_size	**166**	**185**
assortativity	0.0102	0.0267
avg_clustering	**0.0894**	**0.0003**
clustering_entropy	**4.268**	**0.112**
3-edge_motif_count	**214**	**1**
3-edge_motif_zscore	**36.00**	−7.03
modularity	**0.4491**	**0.3381**
avg_community_cohesion	**0.2704**	**0.0000**
avg_community_density	**1.7981**	**2.5173**

## Data Availability

The data presented in this study are available on request from the corresponding authors. Data are not publicly available due to privacy.
